# A review of the content and psychometric properties of cancer-related fatigue (CRF) measures used to assess fatigue in intervention studies

**DOI:** 10.1007/s00520-022-07305-x

**Published:** 2022-08-24

**Authors:** Rachel Campbell, Renée Bultijnck, Gemma Ingham, Chindhu Shunmuga Sundaram, Joshua F. Wiley, Jasmine Yee, Haryana M. Dhillon, Joanne Shaw

**Affiliations:** 1 University of Sydney, Faculty of Science, School of Psychology, Psycho-oncology Co-operative Research Group (PoCoG), Sydney, NSW Australia; 2 Department of Human Structure and Repair, Ghent University, Ghent, Belgium; 3 Palliative Care Department, Prince of Wales Hospital, Randwick, NSW Australia; 4 Monash University, Faculty of Medicine Nursing and Health Sciences, School of Psychological Sciences and Turner Institute for Brain and Mental Health, Melbourne, VIC Australia; 5 Faculty of Health Sciences, School of Exercise and Sport Science, The University of Sydney, Sydney, NSW Australia

**Keywords:** Cancer-related fatigue, COSMIN, Patient-reported outcome measures, Psychometrics, Measurement

## Abstract

**Purpose:**

Cancer-related fatigue (CRF) is a common and debilitating consequence of cancer and its treatment. Numerous supportive care interventions have been developed to alleviate CRF; however, the diversity of outcome measures used to assess CRF limits comparability of findings. We aimed to evaluate the content and psychometric properties of measures used to assess CRF in interventions targeting fatigue, to inform the selection of suitable measures in future research.

**Methods:**

Included measures were identified from a systematic review of interventions targeting CRF. General characteristics of each measure were extracted, and item content was assessed against domains specified by the National Comprehensive Cancer Network (NCCN) definition of CRF. Psychometric properties were evaluated against COnsensus-based Standards for the selection of heath Measurement INstruments (COSMIN) criteria.

**Results:**

Of 54 measures identified, 25 met inclusion criteria. Seventeen were fatigue-specific and eight a fatigue subscale or single item within a broader measure. Only 14 (56%) were specifically developed for cancer populations. Content coverage according to the NCCN CRF definition ranged from 0 to 75%. Evidence for fulfilment of COSMIN criteria in cancer populations ranged from 0 to 93%, with only five measures meeting > 70% of the COSMIN criteria.

**Conclusion:**

The Piper Fatigue Scale-Revised had good content coverage, but did not comprehensively address COSMIN criteria. The EORTC-FA12 and FACIT/FACT-F had excellent psychometric properties, with each capturing different aspects of fatigue. Ultimately, the choice of CRF measure should be guided by the research question and the CRF domains most relevant to the particular research context.

**Supplementary Information:**

The online version contains supplementary material available at 10.1007/s00520-022-07305-x.

## Introduction

Cancer-related fatigue (CRF) is a common and debilitating consequence of cancer and its treatments, impacting over half of people with cancer [1]. CRF is a side effect of various treatments including chemotherapy, radiation therapy, immunotherapy, and hormone therapy [2], affecting some people for months or years after treatment [3–5]. CRF is qualitatively distinct from fatigue occurring in the general population due to its severity and pervasive impact on daily activities and quality of life [6, 7]. For this reason, comprehensive assessment of CRF requires measures specifically developed for, and validated in, cancer populations.

The National Comprehensive Cancer Network (NCCN) defines CRF as “a distressing, persistent, subjective sense of physical, emotional, and/or cognitive tiredness or exhaustion related to cancer and/or cancer treatment not proportional to recent activity, and interferes with usual functioning” [8]. Thus, CRF is conceptualised as a multidimensional symptom comprising physical, emotional, and cognitive aspects, as well as a subjective phenomenon, appraised by the individual via self-report. Although many patient-reported outcome measures assess fatigue, some conceptualise CRF as multidimensional in line with the NCCN definition, whereas others conceptualise CRF as a unidimensional construct. No “gold standard” instrument exists to measure CRF, and there is a lack of guidance on which of the available measures to use for different clinical contexts and research purposes [9].

To address the significant burden of CRF on cancer patients and survivors, numerous psychosocial, lifestyle, and pharmacological interventions have been evaluated in an attempt to alleviate CRF [10]. A recent systematic review identified 30 review articles that collectively report on over 300 interventions targeting CRF [11]. However, there was large variability in the patient-reported outcome measures used to assess CRF across interventions, each likely varying in their content and ability to accurately identify and detect changes in CRF. This is problematic as it limits the comparability of findings across studies, precluding robust conclusions about the relative effectiveness of different interventions to inform clinical practice.

Although previous reviews have identified and examined CRF measures [12–14], none evaluated whether the content of the items within each measure adequately captured CRF and only a limited number of psychometric properties were evaluated. This review aims to comprehensively examine both the content and psychometric properties of patient-reported outcome measures used to assess CRF in intervention studies aimed at alleviating CRF, to inform the selection of suitable measures in future research. This review builds on previous reviews by (i) focussing exclusively on CRF measures that have been used as a primary or secondary outcome measure in intervention studies targeting CRF, (ii) evaluating the content of CRF measures against the NCCN definition of CRF, and (iii) using the COnsensus-based Standards for the selection of health Measurement INstruments (COSMIN) criteria [15], which provide international consensus-based standards for assessing the measurement properties of patient reported outcome measures.

## Method

### Selection criteria

Patient-reported outcome measures were identified from a systematic review of systematic reviews and meta-analyses of interventions targeting CRF ([11] see Supplementary File 1 for the full review search strategy). Any measure identified in this systematic review as a primary or secondary outcome measure used to assess fatigue in an intervention study was eligible for inclusion. Two reviewers (RC as the primary reviewer and JY or RB as the secondary reviewer alternately) screened measures to assess whether they met inclusion criteria. Fatigue measures were included if they were self-report, available in English, and had published reports detailing their development and validation. Both fatigue-specific and global health status measures were included if they produced a separate fatigue score and had been specifically used in an intervention study to assess fatigue. If eligible measures had multiple versions (e.g. EORTC FA-13/EORTC FA-12), we listed the most recently developed version but also evaluated earlier versions in the analysis.

### Data extraction

The original development papers were identified for each included measure by searching the PROQOLID database [16], Medline and PsycInfo databases, and reference lists of studies citing each measure. If the original development paper was unclear or not identifiable, the measure developers were contacted for the correct reference. Data extraction occurred between August 2020 and May 2021. The following general characteristics were extracted for each measure according to information provided in the original development papers and other key references: fatigue-specific measure or a subscale/item within a broader measure, population measure was developed for, number of items, number and type of fatigue domains covered, recall period, availability of cancer-specific cut-off scores, and availability of cancer-specific minimally important differences.

## Analysis

The analysis of included patient-reported outcome measures consisted of two phases: (1) content evaluation and (2) appraisal of psychometric properties.

### Content evaluation

Items from included measures were assessed according to whether their content captured the eight aspects specified by the NCCN definition of CRF [8]. Specifically, we assessed whether the item content of each measure captured fatigue that is (1) related to cancer and/or cancer treatment; (2) distressing; (3) persistent; and captures (4) physical, (5) emotional, and (6) cognitive aspects of fatigue; as well as fatigue (7) that is not proportional to recent activity; and (8) interferes with usual functioning. Importantly, item content was assessed according to whether the reviewer team considered items to capture NCCN-specified domains, and did not take into account the domain labels as specified originally by the questionnaire developers. See Table S1 in the supplementary file for criteria used by reviewers to assess item content against the NCCN specified domains.

### Appraisal of psychometric properties

A search for psychometric validation data in cancer populations was conducted for each measure by reviewing all validation studies citing the original development paper. We also searched MEDLINE and PsychInfo databases using the following terms: *name of specific measure* + “development” or “validation” or “validity” or “reliability” or “psychometric”. The psychometric properties of included measures were assessed against international COnsensus-based Standards for the selection of health Measurement INstruments (COSMIN) [15, 17]. These criteria were developed through a comprehensive four-round Delphi study with international experts (psychologists, epidemiologists, statisticians, and clinicians) [15]. Each measure was evaluated against the following COSMIN criteria: item generation (literature, patient interviews, clinician/expert interviews), item reduction (percentage of missing total scores, percentage of missing items, factor analysis), reliability (internal consistency and test retest), content validity, item total correlations, convergent/divergent validity, known group validity, cross-cultural validity, and responsiveness.

Extraction of measure characteristics and analysis of the content and psychometric properties of each measure were conducted independently by two members of the reviewer team (RC, RB, GI, CS, JW, JY, HD, or JS). Any discrepancies were resolved through team discussion until consensus was reached. The percentage of (i) NCCN CRF domains covered and (ii) COSMIN criteria addressed was calculated for each included measures. For the purpose of this review, measures were considered to have “acceptable” or “good” content coverage and psychometric properties if they captured 50% or 70% of NCCN-specified CRF domains and COSMIN criteria, respectively.

## Results

### Characteristics of the measures

**Fig. 1:**
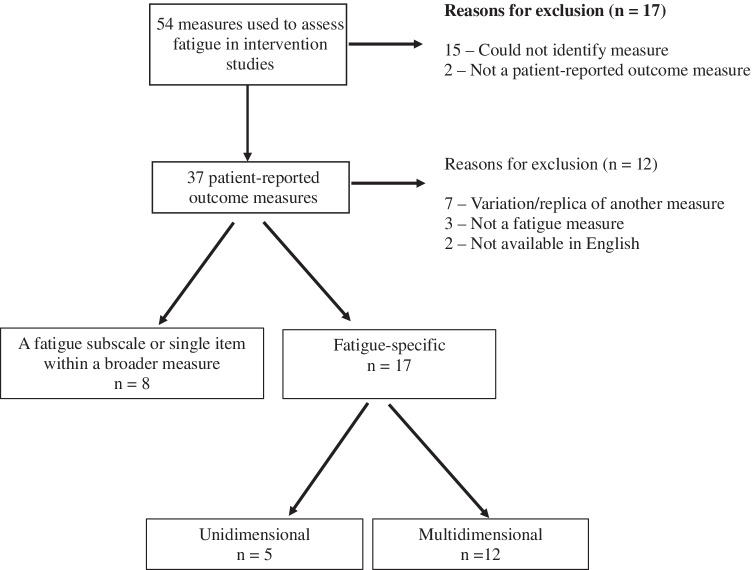
Overview of included measures

Of the 54 measures identified, 25 were eligible for inclusion (Fig. 1). Characteristics of included measures are provided in Table 1. Included measures were developed between 1981 and 2012. Fourteen (56%) were developed for use in cancer populations, eight (32%) for other patient populations with chronic medical conditions, one (4%) for psychiatric patients, and two (8%) for the general population. Eight (32%) were a fatigue subscale or single item within a broader measure, and 17 (68%) were fatigue-specific measures. Of the fatigue-specific measures, five (29%) were unidimensional and 12 (71%) multidimensional. The number of items and domains in each measure was variable, with items ranging from 1 to 40 and domains ranging from 1 to 5. Nine (36%) measures had cancer-specific cut-off scores available to facilitate identification of clinically significant levels of fatigue. Cancer-specific minimally important differences (i.e. the smallest change in a fatigue score individuals identify as meaningful) had been established for eight (32%) measures to aid interpretation of clinically meaningful changes in scores.

**Table 1:** Characteristics of included patient-reported outcome measures

Name of measure (year, country of development)	Fatigue-specific or subscale/item within a broader measure	Population measure was developed for	# items	# Number/type of fatigue domains covered as specified by measure developers	Recall period	Response scale	Availability of cancer-specific cut-offs	Availability of cancer-specific MIDs	Total NCCN domains covered (%)	Total COSMIN criteria met (%)
FACIT/FACT-F (1997, USA)	Subscale within measure	Cancer	40 (13 fatigue-specific)	1 (physical)	Past 7 days	5-point Likert	Y	Y	2 (25)	12 (86)
PFS-R (1998, USA)	Fatigue-specific	Cancer	22	4 (behavioural/ severity, affective meaning, sensory, cognitive/mood)	Now	0–10 numeric rating scale	Y	N	6 (75)	5 (29)
EORTC-QLQ-C30* (1993, 13 countries)	Subscale within measure	Cancer	30 (3 fatigue- specific)	1 (physical)	Past wk	4-point Likert	Y	Y	1 (12.5)	8 (57)
BFI* (1999, USA)	Fatigue-specific	Cancer	9	1 (severity/function)	Past wk and past 24 h	0–10 numeric rating scale	Y	N	3 (38)	5 (29)
POMS-Fatigue (1995, USA)	Subscale within measure	Outpatient psychiatric patients	37 (5 fatigue-specific)	1 (fatigue)	Now	5-point Likert	Y	Y	1 (12.5)	4 (29)
MFI (1994, The Netherlands)	Fatigue-specific	General population	20	5 (general, physical, reduced motivation, reduced activity, mental fatigue)	Lately	5-point Likert	N	Y	3 (38)	8 (57)
SCFS-6* (1997, USA)	Fatigue-specific	Cancer	6	2 (physical, perceptual)	Past 2–3 days	5-point Likert	N	Y	2 (25)	11 (79)
LFS (1990, USA)	Fatigue-specific	Heterogeneous patient populations	18	2 (fatigue, energy)	Now	11-point Likert	N	N	2 (25)	6 (43)
FSI (1998, USA)	Fatigue-specific	Cancer	14	3 (fatigue intensity, duration, interference)	Past wk/now	11-point Likert	N	N	3 (38)	11 (79)
MFSI-SF (2004, USA)	Fatigue-specific	Cancer	30	5 (general, physical, emotional, mental fatigue, vigour)	Past 7 days	5-point Likert	N	Y	4 (50)	10 (71)
CIS (1994, The Netherlands)	Fatigue-specific	Chronic fatigue syndrome	20	4 (subjective fatigue, concentration, motivation, physical activity)	Past 2 wks	7-point Likert	N	N	3 (38)	3 (21)
FSS* (1989, USA)	Fatigue-specific	MS, systemic erythematosus, and CFS	9	1 (fatigue)	Past 2 wks	7-point Likert	N	N	3 (38)	4 (29)
CaFS (2000, Japan)	Fatigue-specific	Cancer	15	3 (physical, affective, cognitive)	Now	5-point Likert	Y	N	3 (38)	8 (57)
SF-36* (1998, USA)	Subscale within measure	General population	36 (4 fatigue-specific)	1 (vitality) (energy/fatigue)	Past 4 wks	6-point Likert	N	N	2 (25)	4 (29)
CFQ (1993, UK)	Fatigue-specific	CFS/ME	11	2 (physical, mental)	Last month	4-point Likert	N	N	2 (25)	0 (0)
EORTC QLQ-FA12 (2005, Europe)	Fatigue-specific	Cancer	12	5 (physical, emotional, cognitive, interference, social sequelae)	Past wk	4-point Likert	Y	N	4 (50)	13 (93)
FSC* (1981, The Netherlands)	Fatigue-specific	Cancer	30	4 (physical, mental, malaise, psychological complaints)	Not specified	Yes/no	N	N	3 (38)	2 (14)
Single item NRS** (original development paper/source unclear)	Fatigue-specific	Cancer and other chronic disease populations	1	1(fatigue)	Now	11-point horizontal scale	Y	N	0	3 (21)
ESAS (1991, Canada)	1 item within measure	Cancer and palliative care patients	10 (1 fatigue- specific)	1 (tiredness)	Now	0–10 numeric rating scale	Y	Y	1 (12.5)	9 (64)
RSCL (1983, The Netherlands)	Subscale within measure	Cancer	39 (1 fatigue-specific)	1 (tiredness)	Past 7 days	4-point Likert	N	N	1 (12.5)	8 (57)
PROMIS F-SF (2004, USA)	Fatigue-specific	General population and patients with chronic disease	7	1 (fatigue)	Past 7 days	5-point Likert	N	Y	4 (50)	8 (57)
PSEFSM (2012, USA)	Fatigue-specific	Cancer	6	1 (self-efficacy for self-management of fatigue)	Not specified	11-point scale	N	N	2 (25)	7 (50)
MFIS (1994, Canada)	Fatigue-specific	MS	21	3 (physical, cognitive, psychosocial)	Past 4 wks	5-point Likert	N	N	4 (50)	0 (0)
NHP (1981, UK)	Subscale within measure	General population and patients with chronic disease	38 (3 fatigue-specific)	1 (energy)	Now	Yes/no	N	N	2 (25)	0 (0)
CRDQ (1987, Canada)	Subscale within measure	Respiratory tract/pulmonary disease	20 (4 fatigue-specific)	1 (fatigue)	Past 2 wks	7-point Likert	N	N	1 (12.5)	0 (0)

^*^May incur cost or permission required for use

^**^Limited language versions available; all other measures available in multiple languages

*BFI*, Brief Fatigue Inventory; *CaFS*, Cancer Fatigue Scale; *CFQ*, Chalder Fatigue Scale; *CFS*, Chronic Fatigue Syndrome; *CIS*, Checklist Individual Strength; *CRDQ*, Chronic Respiratory Disease Questionnaire; *EORTC QLQ-FA12*, European Organisation for Research and Treatment of Cancer Quality of Life Questionnaire Cancer-Related Fatigue; *EORTC-C30*, European Organisation for Research and Treatment of Cancer Core Quality of Life Questionnaire; *ESAS*, Edmonton Symptoms Assessment System; *FACIT-F*, Functional Assessment of Chronic Illness Therapy-Fatigue; *FSC*, Fatigue Symptom Checklist; *FSI*, Fatigue Symptom Inventory; *FSS*, Fatigue Severity Scale; *LFS*, Lee Fatigue Scale; *ME*, Myalgic encephalomyelitis; *MFI*, Multidimensional fatigue inventory; *MFIS*, Modified Fatigue Impact Scale; *MFSI-SF*, Multidimensional Fatigue Symptom Inventory Short Form; *MID*, Minimally Important Difference; *MS*, Multiple sclerosis; *N*, No; *NHP*, Nottingham Health Profile; *NRS*, Numerical Rating Scale; *R-PFS*, Piper Fatigue Scale (revised); *POMS SF*, Profile of Mood States Short Form; *PROMIS*, Patient-Reported Outcomes Measurement Information System; *PSEFSM*, Measurement of Perceived Self-efficacy for Fatigue Self-management; *RSCL*, Rotterdam Symptom Checklist; *SCFS*, Schwartz Cancer Fatigue Scale; *SF-36*, 36-Item Short Form Survey; *wk(s)*, week(s); *Y*, Yes.

## Content evaluation against NCCN CRF definition

Fatigue-specific items from the included measures were assessed against whether they captured the eight aspects specified by the NCCN definition of CRF (Table 2). None of the measures captured all aspects of the NCCN definition. Physical, emotional, and cognitive fatigue were the most frequently assessed domains, captured by more than half the measures. In contrast, very few measures assessed whether fatigue was distressing, persistent, disproportionate to recent activity, and related to cancer and/or cancer treatment. Only five measures (Piper Fatigue Scale-Revised, Patient Reported Outcomes Measurement Information System [PROMIS] Fatigue Short Form, Multidimensional Fatigue Symptom Inventory Short Form, Modified Fatigue Impact Scale, European Organisation for Research and Treatment of Cancer Quality of Life Questionnaire Cancer-Related Fatigue) covered at least 50% of the conceptual domains specified by the NCCN definition of CRF. Five measures (European Organisation for Research and Treatment of Cancer Quality of Life Questionnaire, Rotterdam Symptom Checklist, Edmonton Symptom Assessment Scale, Profile of Mood States-Fatigue, and the Chronic Respiratory Disease Questionnaire) assessed only one of the NCCN-specified domains; one measure (Numeric Rating Scale) did not assess any of the NCCN-specified aspects of CRF.

**Table 2:**
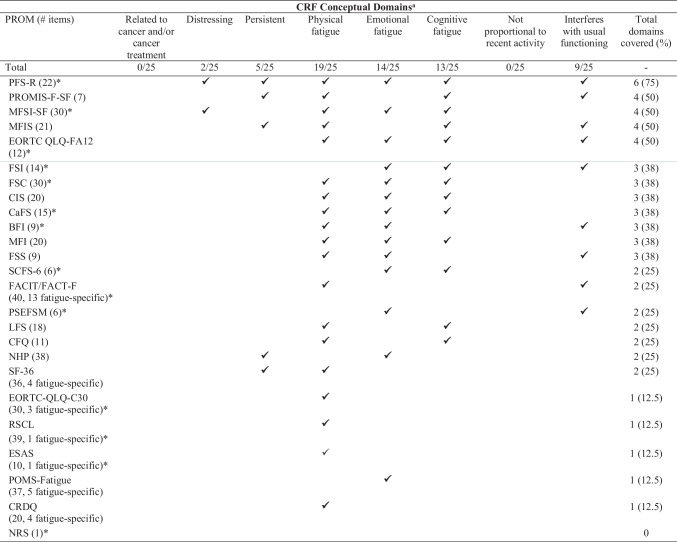
Evaluation of content of the patient-reported outcome measures used to assess CRF in intervention studies assessed against conceptual domains specified by the NCCN CRF definition

✓—Present

^*^These measures were developed specifically for cancer populations; their content did not explicitly note assessment of fatigue in relation to cancer and/or cancer treatment

Only fatigue-specific items within each measure were evaluated against the NCCN-specified conceptual domains

^a^National Comprehensive Cancer Network (NCCN) CRF definition: Cancer-related fatigue is a distressing, persistent, subjective sense of physical, emotional, and/or cognitive tiredness of exhaustion related to cancer and or cancer treatment that is not proportional to recent activity and interferes with usual functioning

Measures above the grey line were deemed to have acceptable/good content coverage (i.e. covered at least 50% of NCCN specified domains)

*BFI*, Brief Fatigue Inventory; *CaFS*, Cancer Fatigue Scale; *CFQ*, Chalder Fatigue Scale; *CIS*, Checklist Individual Strength; *CRDQ*, Chronic Respiratory Disease Questionnaire; *EORTC QLQ-FA12*, European Organisation for Research and Treatment of Cancer Quality of Life Questionnaire Cancer-Related Fatigue; *EORTC-C30*, European Organisation for Research and Treatment of Cancer Core Quality of Life Questionnaire; *ESAS*, Edmonton Symptoms Assessment System; *FACIT-F*, Functional Assessment of Chronic Illness Therapy-Fatigue; *FSC*, Fatigue Symptom Checklist; FSI, Fatigue Symptom Inventory; *FSS*, Fatigue Severity Scale; *LFS*, Lee Fatigue Scale; *MFI*, Multidimensional fatigue inventory; *MFIS*, Modified Fatigue Impact Scale; *MFSI-SF*, Multidimensional Fatigue Symptom Inventory Short Form; *NHP*, Nottingham Health Profile; *NRS*, Numerical Rating Scale; *R-PFS*, Piper Fatigue Scale (revised); *POMS SF*, Profile of Mood States Short Form; *PROM*, Patient reported outcome measure; *PROMIS-F-SF*, Patient-Reported Outcomes Measurement Information System Fatigue Short Form; *PSEFSM*, Measurement of Perceived Self-efficacy for Fatigue Self-management; *RSCL*, Rotterdam Symptom Checklist; *SCFS*, Schwartz Cancer Fatigue Scale; *SF-36*, 36-Item Short Form Survey

## Assessment of psychometric properties against COSMIN criteria

Table 3 displays the assessment of each measure against COSMIN criteria for item generation and item reduction. Only eight (32%) measures generated items through a process of literature review and interviewing both people with cancer and clinicians/experts. With respect to item reduction, percentage of missing total scores was not reported for any measures, percentage of missing items was only reported for four measures (16%), and factor analysis was reported for 16 measures (64%). Six measures (24%) did not include people with cancer in the item generation or item reduction phases; five of these measures were developed for use in the general population or other patient groups.

**Table 3:** Evaluation of patient-reported outcome measures (PROMs) used to assess CRF in intervention studies against evidence of COSMIN criteria in cancer populations

COSMIN criteria							
	Item generation			Item reduction			
				Missing data			
PROM (# items)	Literature	Patient interviews	Clinician/expert interview	% of missing total scores	% of missing items	Factor analysis	Total criteria met (%)
Total	12/25	10/25	11/25	0/25	4/25	16/25	-
SCFS-6 (6)	✓	✓	✓		✓	✓	5 (83)
EORTC QLQ-FA12 (12)	✓	✓	✓		✓	✓	5 (83)
CaFS (15)	✓	✓	✓			✓	4 (67)
PROMIS-F-SF (7)	✓	✓	✓			✓	4 (67)
PSEFSM (6)	✓	✓	✓			✓	4 (67)
FACIT/FACT-F (40)	✓	✓	✓			✓	4 (67)
PFS-R (22)	✓		✓			✓	3 (50)
MFI (20)	✓				✓	✓	3 (50)
FSI (14)	✓	✓	✓				3 (50)
MFSI-SF (30)	✓		✓			✓	3 (50)
ESAS (10)	✓	✓	✓				3 (50)
RSCL (39)		✓	✓			✓	3 (50)
LFS (18)					✓	✓	2 (33)
FSC (30)	✓	✓					2 (33)
BFI (9)						✓	1 (17)
POMS-Fatigue (37)						✓	1 (17)
FSS (9)						✓	1 (17)
CIS (20)						✓	1 (17)
EORTC-QLQ-C30 (30)						✓	1 (17)
SF-36 (36)							0
CFQ (11)							0
NRS (1)							0
MFIS (21)							0
NHP (38)							0
CRDQ (20)							0

✓—Present

*BFI*, Brief Fatigue Inventory; *CaFS*, Cancer Fatigue Scale; *CFQ*, Chalder Fatigue Scale; *CIS*, Checklist Individual Strength; *CRDQ*, Chronic Respiratory Disease Questionnaire; *COSMIN* COnsensus-based Standards for the selection of health Measurement Instruments; *EORTC QLQ-FA12*, European Organisation for Research and Treatment of Cancer Quality of Life Questionnaire Cancer-Related Fatigue; *EORTC-C30*, European Organisation for Research and Treatment of Cancer Core Quality of Life Questionnaire; *ESAS*, Edmonton Symptoms Assessment System; *FACIT-F*, Functional Assessment of Chronic Illness Therapy-Fatigue; *FSC*, Fatigue Symptom Checklist; FSI, Fatigue Symptom Inventory; *FSS*, Fatigue Severity Scale; *LFS*, Lee Fatigue Scale; *MFI*, Multidimensional Fatigue Inventory; *MFIS*, Modified Fatigue Impact Scale; *MFSI-SF*, Multidimensional Fatigue Symptom Inventory Short Form; *NHP*, Nottingham Health Profile; *NRS*, Numerical Rating Scale; *R-PFS*, Piper Fatigue Scale (revised); *POMS SF*, Profile of Mood States Short Form; *PROM*, Patient-reported outcome measure; *PROMIS*, Patient-Reported Outcomes Measurement Information System Fatigue Short Form; *PSEFSM*, Measurement of Perceived Self-efficacy for Fatigue Self-management; *RSCL*, Rotterdam Symptom Checklist; *SCFS*, Schwartz Cancer Fatigue Scale; *SF-36*, 36-Item Short Form Survey

Table 4 displays an overview of the psychometric properties of included measures evaluated in cancer populations. The most commonly addressed COSMIN criteria were evidence for internal consistency (19 [76%] measures) and convergent/divergent validity (18 [72%] measures). In contrast, very few measures had documented evidence of content validity (7 [28%] measures) and item total correlations (6 [24%] measures). Thirteen (52%) measures had been translated into languages other than English and validated cross-culturally in a cancer population. Only three measures (European Organization for Research and Treatment of Cancer [EORTC] Quality of Life—Fatigue questionnaire [EORTC QLQ-FA12], Functional Assessment of Chronic Illness Therapy-Fatigue [FACIT/FACT-F] and Fatigue Symptom Inventory) had evidence for all eight psychometric properties. Four measures (Chalder Fatigue Scale, Modified Fatigue Impact Scale, Nottingham Health Profile and Chronic Respiratory Disease Questionnaire) had no psychometric validation data documented in cancer populations. Overall, only 12 (48%) of the 25 measures met at least 50% of the COSMIN criteria, and four (16%) did not fulfil any of the COSMIN criteria.

**Table 4:**
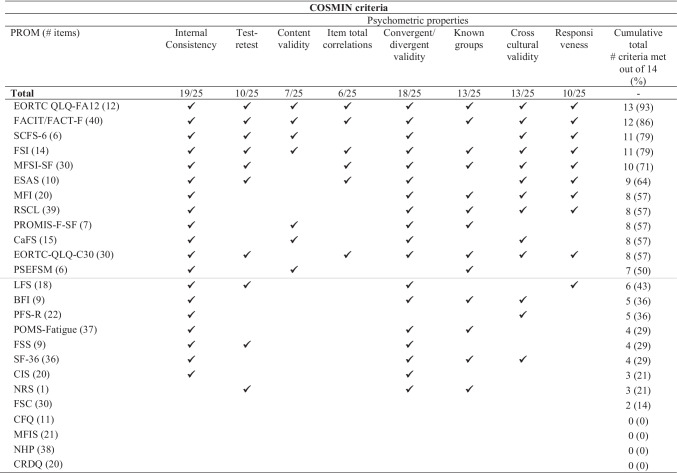
Evaluation of content of the patient-reported outcome measures used to assess CRF in intervention studies assessed against conceptual domains specified by the NCCN CRF definition

✓—Present

Measures above the grey line were deemed to have acceptable/good evidence of psychometric properties in cancer populations (i.e. fulfilled at least 50% of COSMIN criteria)

*BFI*, Brief Fatigue Inventory; *CaFS*, Cancer Fatigue Scale; *CFQ*, Chalder Fatigue Scale; *CIS*, Checklist Individual Strength; *CRDQ*, Chronic Respiratory Disease Questionnaire; *COSMIN*, COnsensus-based Standards for the selection of health Measurement Instruments; *EORTC QLQ-FA12*, European Organisation for Research and Treatment of Cancer Quality of Life Questionnaire Cancer-Related Fatigue; *EORTC-C30*, European Organisation for Research and Treatment of Cancer Core Quality of Life Questionnaire; *ESAS*, Edmonton Symptoms Assessment System; *FACIT-F*, Functional Assessment of Chronic Illness Therapy-Fatigue; *FSC*, Fatigue Symptom Checklist; FSI, Fatigue Symptom Inventory; *FSS*, Fatigue Severity Scale; *LFS*, Lee Fatigue Scale; *MFI*, Multidimensional Fatigue Inventory; *MFIS*, Modified Fatigue Impact Scale; *MFSI-SF*, Multidimensional Fatigue Symptom Inventory Short Form; *NHP*, Nottingham Health Profile; *NRS*, Numerical Rating Scale; *R-PFS*, Piper Fatigue Scale (revised); *POMS SF*, Profile of Mood States Short Form; *PROM*, Patient-reported outcome measure; *PROMIS*, Patient-Reported Outcomes Measurement Information System Fatigue Short Form; *PSEFSM*, Measurement of Perceived Self-efficacy for Fatigue Self-management; *RSCL*, Rotterdam Symptom Checklist; *SCFS*, Schwartz Cancer Fatigue Scale; *SF-36*, 36-Item Short Form Survey

## Discussion

This review evaluated a diverse range of patient-reported outcome measures used to assess CRF in intervention studies. Many of the reviewed measures had poor content coverage of CRF, and less than half met at least 50% of the COSMIN standards for selection of health measurement instruments. Only five (20%) measures had acceptable/good content coverage (≥ 50% of NCCN specified domains), and only 12 (48%) had acceptable/good evidence for psychometric validation (≥ 50% of COSMIN criteria) in cancer populations. This suggests that many measures used as a primary or secondary outcome measure were not fit for purpose for outcome assessment in intervention studies targeting fatigue in cancer populations. This is concerning as their inclusion may have contributed to inaccurate assessment of fatigue and poor-quality data [18, 19].

With regard to the content of measures reviewed, the Piper Fatigue Scale-Revised most comprehensively captured the domains specified by the NCCN definition, covering 75% of domains, respectively. However, in terms of the COSMIN criteria, the Piper Fatigue Scale-Revised only had evidence for approximately one-third (29%) of the criteria. Thus, even though the Piper Fatigue Scale-Revised captured relevant content, further validation work in cancer populations is needed for this measure to be recommended for inclusion in future studies. Four other measures had acceptable content coverage, capturing 50% of NCCN specified domains (i.e. PROMIS Fatigue Short Form, Multidimensional Fatigue Symptom Inventory Short Form, Modified Fatigue Impact Scale, European Organisation for Research and Treatment of Cancer Quality of Life Questionnaire Cancer-Related Fatigue). These measures had evidence for 57%, 71%, 0%, and 93% of the COSMIN criteria, respectively. Following further validation, the brevity of the PROMIS Fatigue Short Form (7 items) in particular may render it useful for inclusion in studies where minimising patient burden is a key consideration (e.g. studies including patients with advanced stage disease or in palliative care). Of note, PROMIS measures are also designed for computer adaptive testing, further minimising respondent burden [20]. Overall, measures developed specifically for cancer populations tended to cover somewhat more of the NCCN-specified content (range 0 –75% of NCCN-specified domains) than measures developed for other populations (range 12.5–50%). In some instances, our assessment of item content did not align with the domain labels as specified by the questionnaire developers. This highlights the importance of researchers carefully reading questionnaire items when selecting measures to ensure item content matches the outcome of interest.

The most psychometrically robust measures were the European Organisation for Research and Treatment of Cancer Quality of Life Questionnaire-Fatigue (EORTC QLQ-FA12) and the Functional Assessment of Chronic Illness Therapy-Fatigue (FACIT/FACT-F) questionnaire, each fulfilling over 80% of the COSMIN criteria in cancer populations. However, both measures are designed to be administered in conjunction with core quality of life measures (i.e. EORTC Quality of Life Questionnaire-Core 30 and Functional Assessment of Cancer Therapy-General), and their length (> 40 items) may limit their use. As such, their inclusion as an outcome measure may be especially useful for researchers wishing to examine quality of life outcomes in addition to fatigue. Further research is needed to examine the benefits and limitations of administering either of their fatigue-specific subscales alone. The NCCN domains covered also differed between these measures with the EORTC QLQ-FA12 assessing physical, emotional, and cognitive aspects of fatigue and the FACIT/FACT-F capturing only physical aspects of fatigue. Meaning, the choice of which measure to use will depend on the aspect of CRF most relevant to the particular research context.

The interpretation of scores generated by CRF measures is facilitated by establishing a minimally important difference (MID), defined as the smallest change in a score considered to be clinically meaningful [21]. The MID is critical for evaluating the effectiveness of interventions aimed at reducing fatigue. Strikingly, only eight (32%) measures had an established cancer-specific MID, rendering it difficult to interpret the clinical relevance of changes in scores generated by the majority of included measures. Similarly, cut-off scores had only been determined for nine (36%) measures to facilitate identification of patients with clinically significant fatigue who would benefit from appropriate intervention. As these attributes are essential for ensuring the usefulness of CRF measures in both research and clinical contexts, future research is needed to establish these characteristics for the majority of measures included in this review. Ideally, this would include determining MIDs for specific cancer and treatment types.

One measure did not assess any of the NCCN-specified domains (Numerical Rating Scale) and four measures (Chalder Fatigue Scale, Modified Fatigue Impact Scale, Nottingham Health Profile & Chronic Respiratory Disease Questionnaire) did not have any psychometric data available in cancer populations. Clearly, these measures are not recommended for use as outcome measures until further work is done to assess their content validity and psychometric properties in cancer populations. Their use as outcome measures in intervention research highlights the need for researchers to actively follow minimum standards for selection of patient-reported outcome measures (e.g. COSMIN [15] and ISOQOL minimum standards [22]) to prevent generation of poor-quality data. Examples of these minimum standards include evidence for reliability, validity (i.e. content validity, construct validity, responsiveness), and interpretability of scores [22] in cancer populations. Peer-reviewed journals could also require evidence patient-reported outcome measures meet these minimum standards for results to be accepted for publication or request a strong justification for the validity and relevance of the chosen CRF measure.

Overall, this review evaluated a wide range of measures used to assess CRF in intervention studies, each varying considerably in content and the number and type of domains covered. This may have resulted, at least partly, from a lack of consensus on an appropriate conceptual framework and agreed definition of CRF [9]. Although the NCCN CRF definition is one of the most commonly used definitions, there is no consensus on a gold standard definition and conceptual framework. This lack of consensus has likely contributed to the proliferation of available measures and the inclusion of such a diverse range of CRF measures in intervention studies. In the absence of a gold standard definition, some caution is warranted when interpreting the present results based on the NCCN definition. Nevertheless, an advantage of our approach is that it enabled comparison of item content across fatigue measures using a single definition. There is a strong need for the development of a consensus-based definition and conceptual framework to guide CRF measure selection in future studies.

Ultimately, the choice of which CRF measure to select will depend on the definition of CRF adopted within a study, the study purpose, and research question, as well as the aspects of fatigue most relevant to the particular intervention or research context. Other key considerations include the purpose of assessment (i.e. for screening or outcome assessment), the relevant timeframe of interest, whether a measure captures other outcomes of interest (e.g. specific symptoms, quality of life). and the existence of interpretation/scoring guidelines (i.e. cut-off scores/MIDs). When choosing between unidimensional and multidimensional CRF measures, there are advantages and disadvantages of both to consider. Unidimensional measures tend to be more limited in their assessment of CRF, whereas multidimensional measures can provide more comprehensive assessment. Unidimensional measures may be appropriate if the main outcome of interest is a single outcome (e.g. physical fatigue or fatigue severity), whereas multidimensional measures are more suitable for exploring intervention impacts on different fatigue domains. For example, an exercise intervention may improve physical fatigue but may not have any effect on emotional fatigue. Finally, unidimensional measures tend to be briefer and less burdensome so may be more suited for repeated longitudinal assessments and contexts where limiting questionnaire length is desirable. Although not the focus of this review, additional considerations especially important when selecting CRF measures for use in clinical practice include ease of administration, completion, and scoring. A future evaluation should focus on the clinical utility of CRF measures and their feasibility for use in clinical practice.

A key strength of this review is the evaluation of a comprehensive range of patient-reported outcome measures identified from 30 review articles reporting over 300 interventions targeting CRF [11]. Although we acknowledge that we have not evaluated every CRF measure, we used a pragmatic approach of reviewing measures used as primary or secondary outcomes to evaluate the effectiveness of CRF interventions identified from a broad review. Nevertheless, this review yields unique insight into whether current self-report measurement approaches in fatigue intervention research are fit for purpose. It is also important to note the broader review the CRF measures we appraised were identified from [11] identified several objective measures used to assess CRF in intervention studies. However, an evaluation of these objective measures was beyond the scope of this review and warrants future attention.

This review had some limitations. First, the COSMIN checklist was only released in 2010, and 24 (96%) of the measures included in this review were developed between 1981 and 2009. It is possible that certain COSMIN criteria may have been addressed during the initial measure development phase, but these details were not reported due to the absence of reporting guidelines. To attempt to counteract this, we not only relied on primary development papers but also searched the literature for more recent development/validation papers. We also acknowledge that new data may have become available after our search for psychometric validation data in cancer populations was completed, so more recently published validation studies may have been excluded. We also limited our evaluation to key COSMIN criteria, and did not examine reports of the face validity or floor and ceiling effects of the CRF measures; future evaluation of these properties may further inform the selection of CRF measures. Moreover, this review did not evaluate the quality of available psychometric data. Consequently, some of the psychometric evidence may be based on validation studies with methodological flaws. In addition, we only assessed evidence from validation papers published in English, possibly excluding relevant validation studies published in other languages. Finally, we adopted the widely used NCCN CRF definition as our external criterion for assessing content coverage of CRF measures. However, we acknowledge there is a lack of a consensus-based definition of CRF in the literature [9] and that other definitions are available [23–26]. If we had adopted an alternative definition of CRF, our findings with regard to content coverage would likely have differed. Furthermore, given the NCCN definition was only released in 2001 and 20 (80%) of the included measures were developed before then, it is perhaps somewhat unsurprising that content coverage was poor for the majority of included measures.

In summary, this review demonstrated the majority of measures used to assess CRF as a primary or secondary outcome measure in intervention studies were not fit for purpose. Based on the content and psychometric appraisal conducted, the EORTC-FA12 and FACT-F/FACIT-F are the most valid measures available for assessing CRF in research contexts. Although the Piper Fatigue Scale-Revised demonstrated good content coverage, further validation work in cancer populations is needed for this measure to be recommended for use. The development of a gold standard definition and conceptual framework of CRF would significantly help to inform and guide CRF measure selection in future studies.

## Supplementary Information

Below is the link to the electronic supplementary material.

Supplementary file1 (PDF 117 KB)

Supplementary file2 (DOCX 13 KB)

## Data Availability

All data generated during this study are included in this published article.
