# Supplementary material for: A review of the content and psychometric properties of cancer-related fatigue (CRF) measures used to assess fatigue in intervention studies
Source: Support Care Cancer. 2022 Aug 24;30(11):8871–83. doi: 10.1007/s00520-022-07305-x (PMC9633540; doi:10.1007/s00520-022-07305-x)
Supplement: Supplementary file 1 — Supplementary file1 (PDF 117 KB) [file 520_2022_7305_MOESM1_ESM.pdf]

Cochrane Search

▼ **Search History** (11)

View Saved

# ▲	Searches	Results	Type	Actions	Annotations
1	cancer.mp. [mp=title, short title, abstract, full text, keywords, caption text]		Advanced	Display Results More	
2	fatigue.mp. [mp=title, short title, abstract, full text, keywords, caption text]		Advanced	Display Results More	
3	intervention.mp. [mp=title, short title, abstract, full text, keywords, caption text]		Advanced	Display Results More	
4	'systematic review'.mp. [mp=title, short title, abstract, full text, keywords, caption text]		Advanced	Display Results More	
5	exercise.mp. [mp=title, short title, abstract, full text, keywords, caption text]		Advanced	Display Results More	
6	medication.mp. [mp=title, short title, abstract, full text, keywords, caption text]		Advanced	Display Results More	
7	psychological.mp. [mp=title, short title, abstract, full text, keywords, caption text]		Advanced	Display Results More	
8	1 and 2 and 3 and 4		Advanced	Display Results More	
9	5 or 6 or 7		Advanced	Display Results More	
10	8 and 9		Advanced	Display Results More	
11	limit 10 to full systematic reviews		Advanced	Display Results More	

Contract

Save | Remove | Combine with: AND | OR

Save All | Edit | View Saved

Advanced Search | Basic Search | Find Citation | Search Fields | Multi-Field Search

1 Resource selected | Hide | Change

EBM Reviews - Cochrane Database of Systematic Reviews 2005 to May 2, 2019

Enter keyword or phrase (* or $ for truncation) — ◉ **Keyword** ○ Author ○ Title ○ Journal — Search

▾ **Limits** *(close)* ☐ Include Multimedia

☐ Withdrawn Records ☐ Full Systematic Reviews ☐ Protocols
☐ New Reviews ☐ Recently Updated Reviews

Additional Limits | Edit Limits

Options

To search Open Access content on Ovid, go to Basic Search.

Print | Email | Export | + My Projects | Keep Selected

☑ All | Range | Clear | 25 Per Page | 1 | Go | Next ›

▼ **Search Information**

You searched:
limit 10 to full systematic reviews

Search terms used:
'systematic review'
cancer
exercise
fatigue
intervention
medication
psychological

Search Returned:
281 text results

☑ 1. [illegible]

Table of Contents
Abstract Reference
Complete Reference

Find Similar
Find Citing Articles

Abstract | My Projects | Annotate

EBM Full Text
Find Full Text
