# Supplementary material for: A review of the content and psychometric properties of cancer-related fatigue (CRF) measures used to assess fatigue in intervention studies
Source: Support Care Cancer. 2022 Aug 24;30(11):8871–83. doi: 10.1007/s00520-022-07305-x (PMC9633540; doi:10.1007/s00520-022-07305-x)
Supplement: Supplementary file 2 — Supplementary file2 (DOCX 13 KB) [file 520_2022_7305_MOESM2_ESM.docx]

| Conceptual domains specified by NCCN CRF definition^a^: | Criteria for conceptual domain to be adequately covered by CRF measures: |
| --- | --- |
| Related to cancer and/or cancer treatment | The instructions or items should explicitly note fatigue is being assessed in relation to cancer and/or cancer treatment. |
| Distressing | Items should explicitly assess distress. Items assessing negative emotions or low mood are not sufficient unless clearly capturing distress. |
| Persistent | Items, time frame of assessment or response options should assess whether fatigue is persistent in nature. Persistent is defined as lasting longer than one week. |
| Physical fatigue | Items should assess physical aspects of fatigue (e.g. feeling weak, experiencing muscle ache, lacking energy). |
| Emotional fatigue | Items should assess emotional aspects of fatigue (e.g. feeling anxious, depressed, hopeless, irritable, lacking motivation). |
| Cognitive fatigue | Items should assess cognitive aspects of fatigue (e.g. experiencing difficulties concentrating, remembering, or thinking clearly). |
| Not proportional to recent activity | Items should explicitly assess whether the fatigue experienced is proportional to recent activity (e.g. increased physical activity, strenuous exercise, lack of sleep). |
| Interferes with usual functioning | Items should explicitly assess whether fatigue interferes with usual functioning (e.g. ability to carry out usual daily activities) |

**Table S1.** Reviewer guide for evaluating item content of CRF measures against conceptual domains specified in the NCCN definition of CRF

^a^National Comprehensive Cancer Network (NCCN) CRF definition: Cancer-related fatigue is a distressing, persistent, subjective sense of physical, emotional, and/or cognitive tiredness of exhaustion related to cancer and or cancer treatment that is not proportional to recent activity and interferes with usual functioning.
